# Sinus node-like pacemaker mechanisms regulate ectopic pacemaker activity in the adult rat atrioventricular ring

**DOI:** 10.1038/s41598-019-48276-0

**Published:** 2019-08-13

**Authors:** Sunil Jit R. J. Logantha, Sanjay R. Kharche, Yu Zhang, Andrew J. Atkinson, Guoliang Hao, Mark R. Boyett, Halina Dobrzynski

**Affiliations:** 1 Division of Cardiovascular Sciences, Faculty of Biology, Medicine and Health, University of Manchester, Manchester, United Kingdom; 2 Lawson’s Health Research Institute, Department of Medical Biophysics, University of Western Ontario, London, ON Canada

**Keywords:** Atrial fibrillation, Atrial fibrillation

## Abstract

In adult mammalian hearts, atrioventricular rings (AVRs) surround the atrial orifices of atrioventricular valves and are hotbed of ectopic activity in patients with focal atrial tachycardia. Experimental data offering mechanistic insights into initiation and maintenance of ectopic foci is lacking. We aimed to characterise AVRs in structurally normal rat hearts, identify arrhythmia predisposition and investigate mechanisms underlying arrhythmogenicity. Extracellular potential mapping and intracellular action potential recording techniques were used for electrophysiology, qPCR for gene and, Western blot and immunohistochemistry for protein expression. Conditions favouring ectopic foci were assessed by simulations. In right atrial preparations, sinus node (SN) was dominant and AVRs displayed 1:1 impulse conduction. Detaching SN unmasked ectopic pacemaking in AVRs and pacemaker action potentials were SN-like. Blocking pacemaker current *I*_f_, and disrupting intracellular Ca^2+^ release, prolonged spontaneous cycle length in AVRs, indicating a role for SN-like pacemaker mechanisms. AVRs labelled positive for HCN4, and SERCA2a was comparable to SN. Pacemaking was potentiated by isoproterenol and abolished with carbachol and AVRs had abundant sympathetic nerve endings. β_2_-adrenergic and M_2_-muscarinic receptor mRNA and β_2_-receptor protein were comparable to SN. In computer simulations of a sick SN, ectopic foci in AVR were unmasked, causing transient suppression of SN pacemaking.

## Introduction

The sinoatrial node or simply sinus node (SN), atrioventricular node (AVN) and His- Purkinje network constitute the cardiac conduction system and are responsible for the initiation and coordinated conduction of the heartbeat. The constituent cardiomyocytes of the conduction system display rhythmic automaticity and SN is the dominant pacemaker in the heart^1^. Histologically discreet atrioventricular ring bundles (AV rings/AVRs), anatomically continuous with the AVN, have been shown to be present in the adult mammalian heart. The myocytes of right and left AVRs have been shown to extend inferiorly from the compact AVN, encircling the atrial orifices of tricuspid and mitral valves respectively, and uniting antero-superiorly at the retroaortic node after the right ring crosses over the penetrating bundle component of the AV conduction axis^2–4^. The right AVR is much more extensive than left^2,3^. The AVRs maintain electrical continuity with atrial myocardium^3,5^, and are separated from ventricles by the AV junctional connective tissue^2,3,6^.

The AVRs are derived from the atrioventricular canal myocardium that also gives rise to the compact AVN^6–9^, and the ion channel expression in AVRs is distinct from atrial tissue, and shares much commonality with SN and AVN^1–3,10^. Like nodal tissues, AVRs abundantly express genes responsible for the pacemaker current *I*_f_, the hyperpolarization-activated cyclic nucleotide-gated channels (*HCN1* and *HCN4*)^2,3^. In rat and rabbit, both right and left AVRs express HCN4 protein, with higher abundance in right AVR than in the left. Compared to atrial myocardium, AVRs in the rat, rabbit and dog express less of the inward rectifier K^+^ channel (*K*_*ir*_2*.1*), voltage gated Na^+^ channel (*Na*_*v*_*1.5*) and connexins (*Cx43*)^2,3,10–12^. Such expression profile is consistent with an early observation by de Carvalho *et al*. of slowing right atrial conduction on reaching the AVR and decrease in action potential conduction velocity on moving progressively closer to tricuspid valve^5^.

Electrophysiologically, the AVRs appear to be a hotbed of ectopic foci, responsible for focal atrial tachycardia that can give rise to atrial fibrillation and flutter and the myocardium of the atrioventricular annuli, and particularly the tricuspid annulus, are common targets for ablation in the treatment of focal atrial tachycardia^13–15^. It has also been shown that accessory atrioventricular muscle bundles can arise from the AVRs^6,16^, forming the atrial origin for re-entrant circuits, which could contribute to ventricular tachycardias due to pre-excitation such as in Wolf-Parkinson-White syndrome. The AVR myocytes respond to adenosine (at doses that reliably induce transient AV block) with a decrease in action potential upstroke velocity, amplitude and duration without altering the maximum diastolic potential (MDP). Such adenosine response is analogous to that observed in the compact AVN and is unlike atrial myocytes where adenosine decreases only the action potential duration (APD)^12^. Focal atrial tachycardia arising due to triggered activity and micro re-entry can be terminated with adenosine, however in ectopic/automatic atrial tachycardia response to adenosine is transient and tachycardia reoccurs and radiofrequency ablation remains the most effective treatment option^15,17^. Experimental data offering mechanistic insights into the initiation and maintenance of ectopic foci in AVRs is lacking.

We have characterised the electrophysiology of AVR myocytes in tissue preparations using multi-electrode array mapping and sharp microelectrode action potential recording techniques. To investigate mechanisms underlying ectopic pacemaking, we asked the question whether voltage- and Ca^2+^-clock pacemaker mechanisms, pivotal for SN pacemaking,^18–20^ have a role to play in AVRs. Is pacemaking in AVRs different from that in the SN? These questions were prompted by our preliminary investigation where we showed that AVRs spontaneously elicit pacemaker action potentials with prominent phase 4 diastolic depolarization (DD)^3^. In the present investigation, we therefore proceeded to identify parameters describing the DD *viz*. duration of DD, amplitude of DD, rate of early DD and studied how these differed from those in the SN. To further investigate the mechanisms controlling ectopic foci, we asked the question whether rate-potentiating stimuli, specifically adrenergic activation, affected ectopic pacemaking in AVR? Using computer simulations we reconnoitred conditions that favoured ectopic foci in the AVR.

Our investigations identified that voltage-clock mechanisms underlie ectopic pacemaking in the rat AVR. Adrenergic activation can initiate as well as potentiate AVR pacemaking. The right atrial myocardium consists of electrophysiologically heterogeneous myocytes with different membrane properties providing discrete opportunities for the initiation of ectopic foci, the basis for focal atrial tachycardias.

## Results

### Action potential heterogeneity in the right atrium

**Figure 1:**
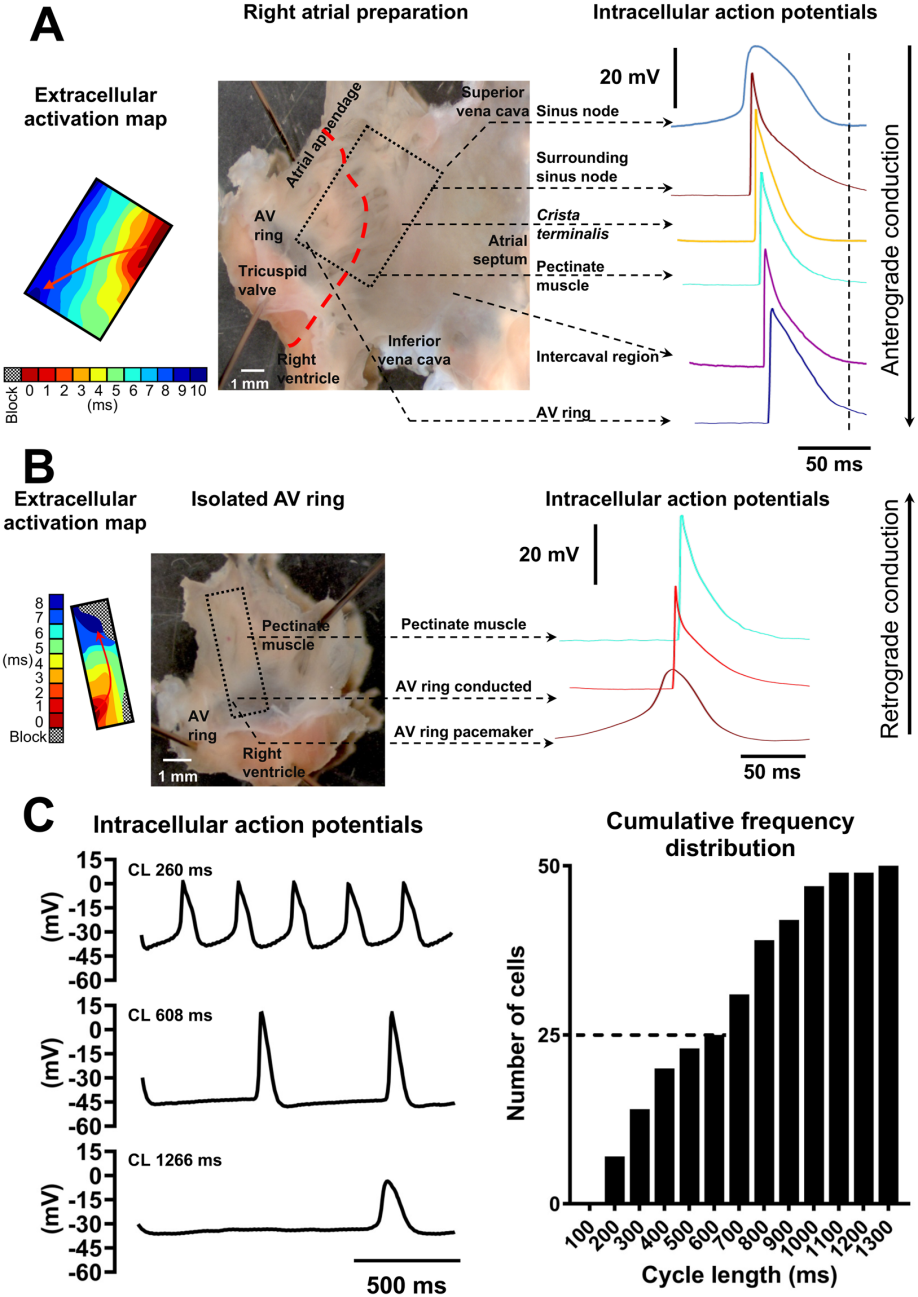
Action potential heterogeneity in the rat right atrium. (**A**) Middle, right atrial preparation with intact sinus node and atrioventricular ring (AV ring). Box denotes mapping array (8 × 8 electrodes) position. Left, extracellular electrical activation map is shown. Red arrow denotes direction of action potential propagation. Right, typical intracellular action potentials are shown arbitrarily shifted to the right and downward for clarity of presentation. (**B**) AV ring isolated by cutting along red dashed line drawn on tissue preparation shown in A. Box denotes mapping array (6 × 10 electrodes) position. Extracellular electrical activation map shown to the left of tissue. Action potentials at AV ring leading pacemaker site, AV ring conducted and pectinate muscles shown to the right. (**C**) AV ring pacemaker action potentials with fast (top), average (middle) and slow (bottom) cycle lengths (CL). Right panel shows cumulative frequency distribution histogram of CLs in AV ring pacemaker cells (n = 50 cells in 21 AV rings). Vertical and horizontal scale bars represent voltage (mV) and time (ms), respectively.

Typical right atrial preparation with intact SN and AVR is shown (Fig. 1A). Multi-electrode mapping consistently identified the initiation of the electrical impulse at anatomical sites to the right of *crista terminalis* at the level of SN (Fig. 1A, left). The electrode array was removed to allow access to the impulse initiation site for sharp microelectrodes. By making multiple microelectrode recordings at the initiation site, the leading pacemaker site was localised to a small zone of ~0.1 mm^2^ in SN. From the leading pacemaker site, electrical activity propagated to *crista terminalis* and thence to pectinate muscles before arriving at AVR (Fig. 1A). Intracellular action potentials were obtained from the leading pacemaker site, area surrounding the leading pacemaker site, *crista terminalis*, pectinate muscle, intercaval region and AVR. AVRs expressed triangular action potentials that differed markedly from other atrial regions (Fig. 1A and data Supplement Table 1). AVR myocytes showed the least negative MDP at −73.4 ± 1 mV, slowest maximal upstroke velocity (dV/dt_max_) of 107.9 ± 6.9 V/s, shortest amplitude at 81.4 ± 1.7 mV and smallest peak potential at 7.6 ± 1.2 mV. The action potential repolarization was slow with the longest APD_10_ of all regions of 5 ± 0.6 ms. APD_50_ of 24.2 ± 1.5 ms and APD_90_ of 68.4 ± 2.4 ms were comparable to atrial cells surrounding SN and in the intercaval region, but significantly longer than *crista terminalis* and pectinate muscles (data Supplement Table 1). Rarely, conducted AVR action potentials with a small yet noticeable DD were encountered; however, neither spontaneous/automatic nor triggered impulse initiation was observed in right AVR in preparations with intact SN.

### Automaticity in the isolated AVR

Electrophysiology of AVR was investigated without the confounding influence of SN in isolated AVR preparations (Fig. 1B). Detaching SN unmasked automaticity i.e. spontaneous electrical activity in AVRs. It is unlikely that the automaticity was caused by the physical process of tissue dissection because automaticity persisted for long periods of time (>60 minutes) with stable cycle lengths that could be reversibly accelerated or decelerated by pharmacological treatments. Additionally, ectopic foci were identified throughout the AVR (n = 21 hearts) by electrical mapping and there wasn’t a pattern of automaticity originating near cut edges. Electrical activity originating in AVR propagated retrogradely to pectinate muscles and towards the *crista terminalis* (Fig. 1B, left). Occasionally, 2–3 ectopic pacemaker sites were encountered per AVR, beating independently of each other, causing refractoriness at wave boundaries and resulting in conduction blocks (Fig. 1B, bottom right corner of the extracellular activation map). Electrical conduction block was also observed in tissue regions that were furthest from AVR ectopic site for e.g. nearer to the *crista terminalis* in Fig. 1B (top right corner of map). Isoproterenol treatment overcame conduction blocks (see later section). Multiple microelectrode recordings made at the AVR initiation site revealed pacemaker action potentials with prominent DD and a SN-like phenotype (Fig. 1B,C). The spontaneous cycle lengths showed large variations with a range of 197.4–1266 ms (Fig. 1C).

**Figure 2:**
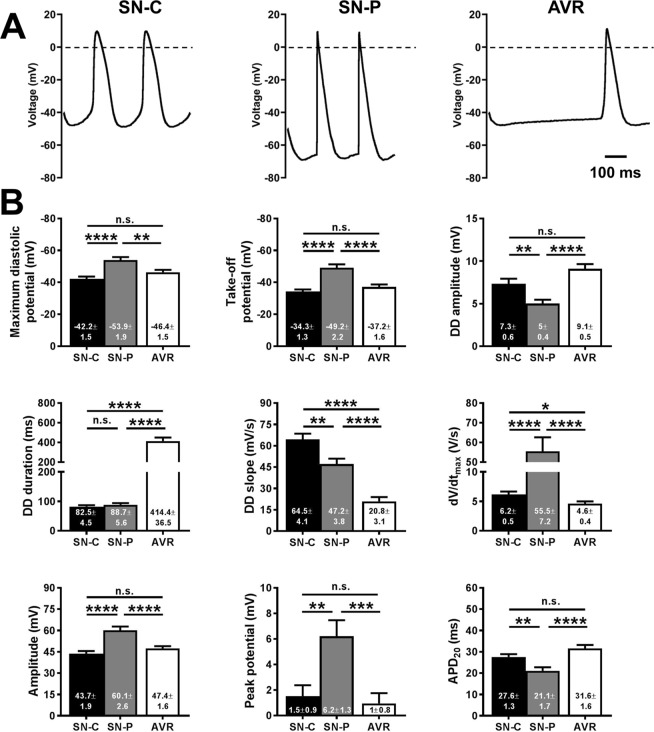
Pacemaker action potential characteristics. (**A**) Typical pacemaker action potentials from sinus node-centre (SN-C), sinus node-periphery (SN-P) and atrioventricular ring (AVR). Scale bar denotes 100 ms. (**B**) Bar charts show action potential parameter measurements in SN-C (n = 21 cells), SN-P (n = 19 cells) and AVR (n = 50 cells). Mean ± s.e.m. values are presented and values shown inside bars. Student’s t-test was used for statistical comparison. *P ≤ 0.05, **P ≤ 0.01, ***P ≤ 0.001, ****P ≤ 0.0001.

The SN is a heterogeneous tissue. Action potentials initiated in the centre of the SN (the leading pacemaker site) propagate to its periphery and then onto the atrial muscle. Action potentials in the SN-centre (SN-C) display less negative MDP, slower dV/dt_max_, smaller amplitude and longer duration than action potentials from SN-periphery (SN-P). We asked the question whether AVRs exhibited such heterogeneity? We proceeded with measuring parameters describing the action potential phenotype and comparing AVR parameters with those of the SN-C and SN-P (Fig. 2). This is the first report of the kind detailing parameter measurements of action potentials recorded in intact SN tissue preparations of the rat. Previous studies have focussed on single cells of rabbit SN.

Measurements of various action potential parameters were taken for AVR (50 cells in 21 hearts), SN-C (21 cells in 9 hearts) and SN-P (19 cells in 10 hearts). Typical pacemaker action potentials in SN-C, SN-P and AVR are shown in Fig. 2A. In isolated AVR the cycle length of pacemaker action potentials was 608.5 ± 41.7 ms (range: 197.4 ms to 1266 ms; Fig. 1C), significantly longer than in SN-C (204.3 ± 5.5 ms; P < 0.0001) and in SN-P (205.9 ± 5.9 ms; P < 0.0001). The MDP and take-off potential (TOP) in AVR were −46.4 ± 1.5 mV and −37.2 ± 1.6 mV, respectively. These values are comparable to those recorded from SN-C: −42.2 ± 1.5 mV and −34.3 ± 1.3 mV, but significantly lower than SN-P: −53.9 ± 1.9 mV (P < 0.01) and −49.2 ± 2.2 mV (P < 0.0001; Fig. 2B). The amplitude of DD was 9.1 ± 0.5 mV in AVRs, similar to SN-C (7.3 ± 0.6 mV), but larger than SN-P (5 ± 0.4 mV; P < 0.0001; Fig. 2B). Consistent with the long cycle length in AVRs, the duration of DD was longest here at 414.4 ± 36.5 ms *vs*. 82.5 ± 4.5 ms in SN-C (P < 0.0001) and *vs*. 88.7 ± 5.6 ms in SN-P (P < 0.0001; Fig. 2B). The slope of DD was 20.8 ± 3.1 mV/s in AVR, significantly lower than SN-C (64.5 ± 4.1 mV/s; P < 0.0001) and SN-P (47.2 ± 3.8 mV/s; P < 0.0001; Fig. 2B). The action potential upstroke was characterised by a dV/dt_max_ of 4.6 ± 0.4 V/s, lower than both SN-C (6.2 ± 0.5 V/s; P < 0.05) and SN-P (55.5 ± 7.2; P < 0.0001; Fig. 2B). The amplitude and peak potential in AVRs was 47.4 ± 1.6 mV and 1 ± 0.8 mV, similar to SN-C (43.7 ± 1.9 mV and 1.5 ± 0.9 mV), but lower than SN-P (60.1 ± 2.6 mV; P < 0.0001 and 6.2 ± 1.3 mV; P < 0.001; Fig. 2B). AVR action potentials exhibited slow early repolarization phase with APD_20_ values of 31.6 ± 1.6 ms analogous to SN-C (27.6 ± 1.3 ms), but longer than SN-P (21.1 ± 1.7 ms; P < 0.0001; Fig. 2B). The late phase repolarization in AVR was the slowest of the three cell types with APD_50_, APD_70_ and APD_90_ values of 61.7 ± 2.5 ms (P < 0.01), 83.5 ± 3.2 ms (P < 0.001) and 219.3 ± 19.1 ms (P < 0.0001), respectively (Fig. 2B). The APD_90_ in AVR was nearly double that in the SN and this was not the case with APD_70_ measurements as these values, in all three cell types, were measured at potentials positive to the TOP and as such excluded the DD. The AVR action potentials were similar in configuration to SN-C with respect to most measured parameters. Deviations were observed only with respect to 2 out of 12 parameters *viz*. the slope and duration of DD.

### Correlation between pacemaker action potential characteristics

**Figure 3:**
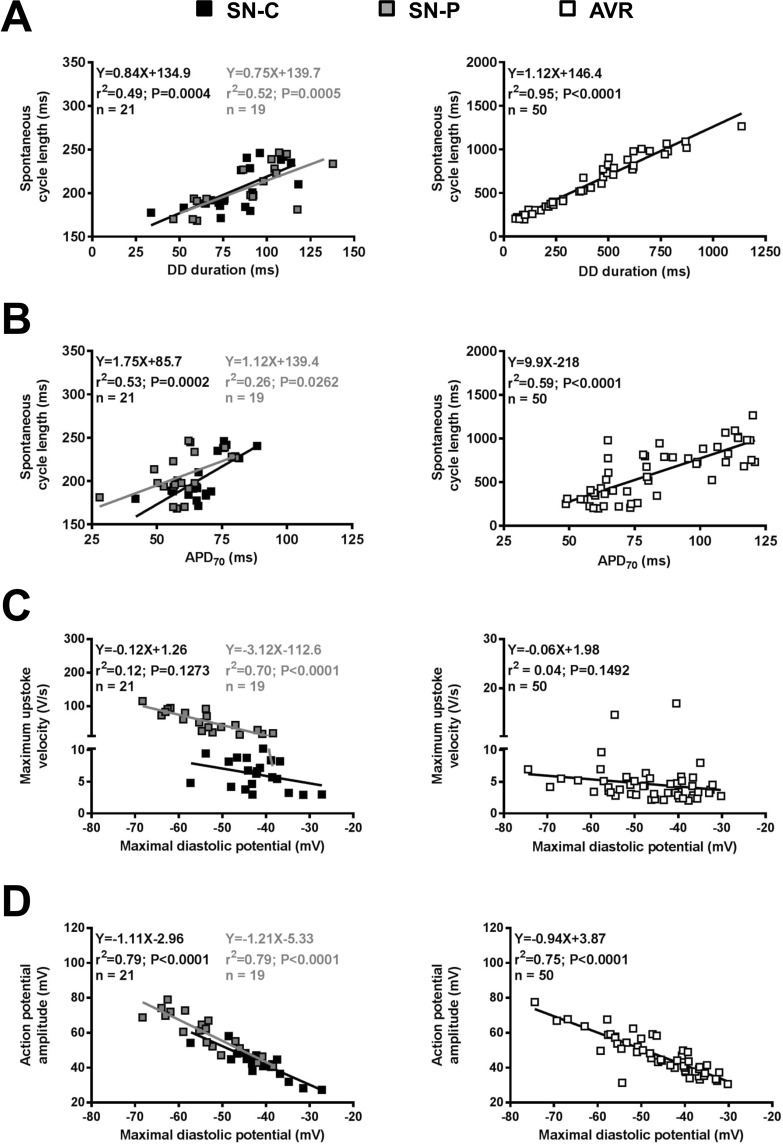
Correlation between pacemaker action potential parameter measurements. (**A**–**D**) correlation plots for sinus node-centre and -periphery (SN-C and SN-P; left plots) and atrioventricular ring (AVR; right plots). Measurements are from 21 SN-C (black squares), 19 SN-P (grey squares) and 50 AVR (white squares) myocytes. Best-fit trend lines were fitted by linear regression and results are shown in inset.

Figure 3 shows the relationship between pacemaker action potential parameters assessed by generating correlation plots and fitting trend lines by linear regression. Parameter measurements from 50 AVR myocytes (21 hearts) were plotted and studied in comparison to 21 SN-C (9 hearts) and 19 SN-P (10 hearts) myocytes. Since the spontaneous cycle length is theoretically determined by the DD duration and APD_70_ (i.e. APD positive to the TOP), relationships between these three measurements were investigated. In AVR pacemaker cells, a significant correlation between DD duration and spontaneous cycle length was observed (r^2^ = 0.95, P < 0.0001), consistent with the behaviour of SN-C (r^2^ = 0.49, P = 0.0004) and SN-P (r^2^ = 0.52, P = 0.0005; Fig. 3A). DD duration is set by the rate of DD (i.e. slope of phase 4) and this rate significantly correlated with spontaneous cycle length in SN-P (r^2^ = 0.25, P = 0.03) and AVR (r^2^ = 0.39, P < 0.0001; data Supplement Fig. 1A). There was no significant correlation between rate of DD and MDP (data Supplement Fig. 1B). APD_70_ and spontaneous cycle length significantly correlated in AVR (r^2^ = 0.59, P < 0.0001), SN-C (r^2^ = 0.53, P = 0.0002) and SN-P (r^2^ = 0.26, P = 0.0262; Fig. 3B). The relationship between MDP and measurements concerned with the upstroke phase of the action potential is shown in Fig. 3C,D. The dV/dt_max_ in SN ranged between 2.9 and 115 V/s and cells with values <10 V/s were categorised as SN-C and the remainder SN-P. In 48/50 AVR cells studied, dV/dt_max_ was <10 V/s and there was no correlation with MDP (Fig. 3C). Action potential amplitude significantly correlated with MDP in SN-C (r^2^ = 0.79, P < 0.0001), SN-P (r^2^ = 0.79, P < 0.0001) and AVR (r^2^ = 0.75, P < 0.0001; Fig. 3D). Significant correlation between MDP and TOP, and TOP and amplitude was observed (data Supplement Fig. 1C,D). Correlation analysis showed that AVR pacemaker cells possess electrophysiological properties quite similar to SN-C. A common ionic mechanism may underlie pacemaking in these two tissues.

### Pacemaker mechanisms in AVR

**Figure 4:**
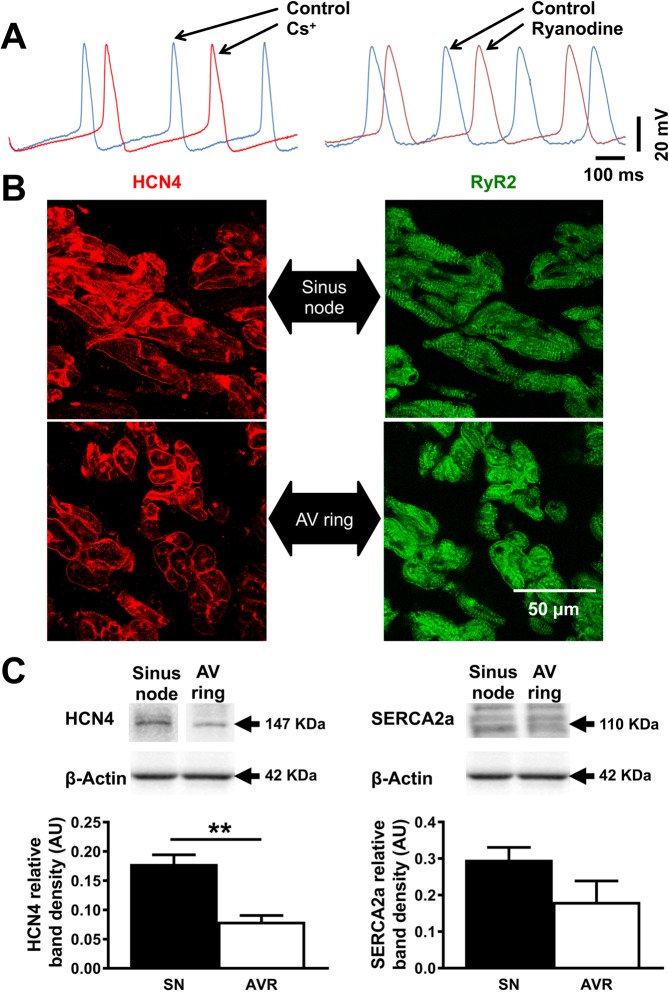
Pacemaker mechanisms in the atrioventricular ring. (**A**) The effects of Cs^+^ and ryanodine on atrioventricular (AV) ring pacemaker action potentials. AV ring pacemaker action potentials at baseline (blue traces), in 2 mM Cs^+^ (left; red trace) and 2 µM ryanodine (right; red trace). Vertical and horizontal scale bars represent voltage (mV) and time (ms), respectively. (**B**) Sinus node and AV ring tissue sections immunolabelled for HCN4 (red signal) and ryanodine receptor (RyR2, green signal). (**C**) Western blots of HCN4 and SERCA2a in sinus node (SN) and AV ring (AVR). Blots cropped from different parts of the same gel, or from different gels are clearly delineated with white space. Student’s t-test was used for statistical comparison. **P ≤ 0.01 and n = 4/5 hearts.

Having ascertained that isolated AVRs can spontaneously initiate SN-like pacemaker action potentials, we investigated the mechanisms responsible for pacemaking (Fig. 4). The DD in SN is a result of synergistic interaction between the membrane voltage-clock and subcellular Ca^2+^-clock^21^. The voltage-clock comprises plasma membrane bound, voltage-dependent ion channels and their corresponding ionic currents, chief amongst them being the HCN or “funny current”, *I*_f_. To confirm the functional contribution of *I*_f_ in AVR automaticity, the effects of Cs^+^ (2 mM CsCl), a reliable *I*_f_ blocker^22^, was investigated in four spontaneously beating AVRs. Pacemaker action potentials were recorded under control conditions and subsequently Cs^+^ was introduced (Fig. 4A). Blocking *I*_f_ with Cs^+^ diminished beating rates and the action potential cycle length of 564.9 ± 87.8 ms in control was prolonged to 771.4 ± 101.9 ms in Cs^+^ (P < 0.05). Consistent with this observation, Cs^+^ significantly diminished the slope of DD (control: 17.2 ± 2.7 mV/s → Cs^+^: 14.7 ± 2.9 mV/s; P < 0.05). The MDP and action potential parameters including TOP, dV/dt_max_, amplitude and duration remained unaffected in Cs^+^. Immunolabelling for HCN4 protein in AVR tissue sections revealed positive labelling throughout the AVR, along the outer cell membranes, similar to that in SN (Fig. 4B, red signal)^2,3^. Identically treated adjacent right atrial tissue was HCN4 negative. HCN4 expression was quantified using Western blot and the expression level in AVRs was ~55% less than in SN (P < 0.01; Fig. 4C).

The Ca^2+^-clock contributes to DD in SN through localised Ca^2+^ release from the sarcoplasmic reticulum via ryanodine receptor (RYR2) gated channels. While some Ca^2+^ is extruded out of the cell by the Na^+^-Ca^2+^ exchanger (NCX1) generating an inward current (*I*_NaCa_), the majority of Ca^2+^ is returned to sarcoplasmic reticulum by sarcoplasmic reticulum Ca^2+^-ATPase (SERCA2a). To assess the possibility of Ca^2+^-clock involvement in AVR, Ca^2+^ release from sarcoplasmic reticulum was disrupted with ryanodine (2 µM) in five spontaneously beating AVRs (Fig. 4A). Action potential cycle length under control conditions of 301.8 ± 51.8 ms was prolonged to 1444 ± 402.6 ms (P < 0.05) in ryanodine. Immunolabelling for RyR2 revealed a punctate pattern of expression adjacent to the outer cell membrane together with random spots and at times striated internal labelling. The labelling pattern in AVR was similar to SN (Fig. 4B, green signal)^3^. SERCA2a levels assessed by Western blot revealed comparable levels in AVR and SN (Fig. 4C). Our investigations of pacemaker mechanisms and expression of associated proteins in AVRs showed stark similarities *vs*. SN.

### Autonomic modulation of automaticity

**Figure 5:**
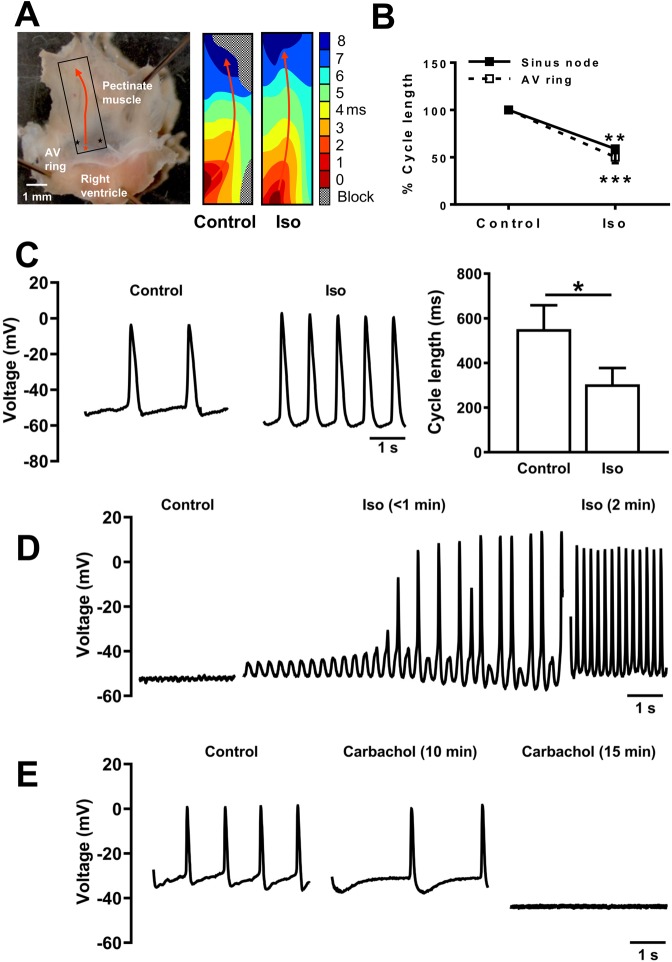
Autonomic modulation of pacemaker activity. (**A**) Typical isolated atrioventricular ring (AV ring) preparation. Box denotes mapping array (6 × 10 electrodes) position, red star and arrow, initiation site and direction of action potential propagation. Scale bar denotes 1 mm. Isoproterenol (Iso) unmasked additional ectopic pacemakers (black stars) and overcame conduction blocks. Electrical activation maps for control and Iso shown. (**B**) Cycle length data from mapping array recordings under control and in iso for sinus node (black squares and continuous line; n = 3) and isolated AV ring (white squares and dashed line; n = 5). Two-way ANOVA with Sidak multiple comparisons post-test was used for statistical comparison. Black asterisks denote significant difference *vs*. respective control. (**C**) Representative records of AV ring pacemaker action potentials under control and in iso. Action potential cycle length is shown in bar chart (n = 4 cells in 4 AV rings). (**D**) Iso induced pacemaker action potentials in a quiescent AV ring. (**E**) Carbachol (0.05 µM) abolished pacemaking in AV ring. *P ≤ 0.05, **P ≤ 0.01, ***P ≤ 0.001. For action potential records in (**C**–**E**) scale bars represent 1 s.

**Figure 6:**
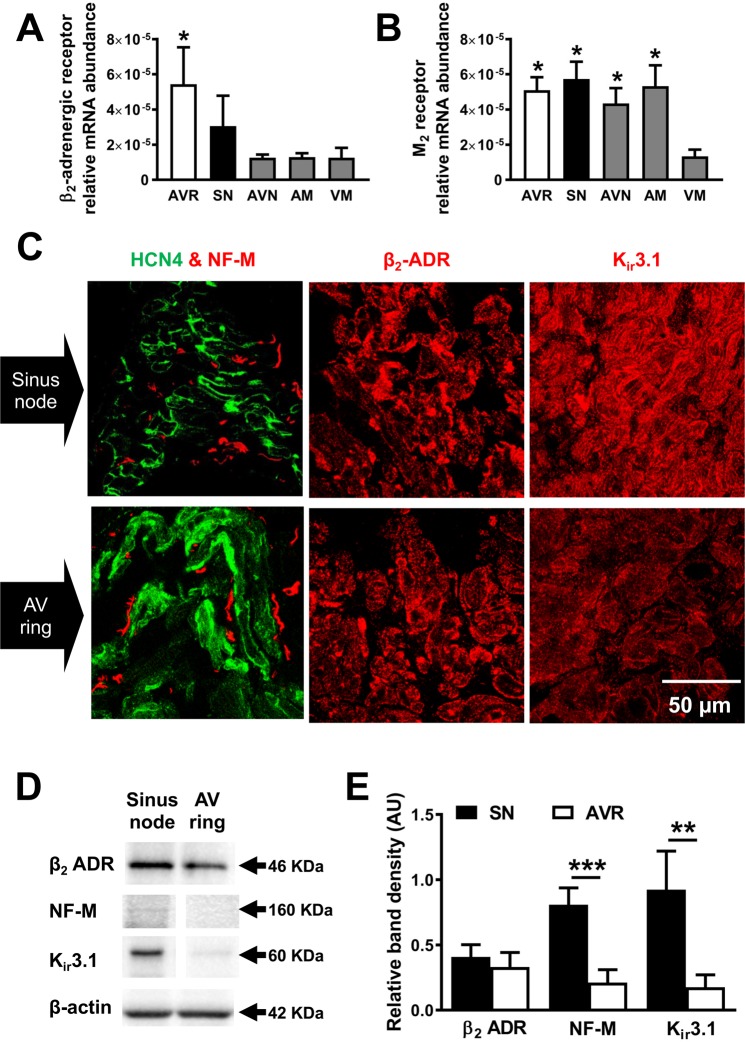
Adrenergic and muscarinic receptor gene and protein expression. (**A**,**B**) β_2_-adrenergic receptor (β_2_-ADR) and M_2_-muscarinic receptor in atrioventricular ring (AVR), sinus node (SN), atrioventricular node (AVN), atrial muscle (AM) and ventricular muscle (VM). *P ≤ 0.05 *vs*. VM; n = 12 hearts. (**C**) Representative SN and AV ring sections labelled with HCN4 (green), neurofilament-M (NF-M; red), β_2_-ADR and K_ir_3.1 (n = 5 hearts). Scale bar denotes 50 µm. (**D**) Western blots of β_2_-ADR, NF-M, K_ir_3.1 and β-actin in sinus node and AV ring. Blots cropped from different parts of the same gel, or from different gels are clearly delineated with white space. (**E**) Mean band densities relative to β-actin. Student’s t-test was used for statistical comparison and **P ≤ 0.01, ***P ≤ 0.001; n = 4/5 hearts.

Figures 5, 6 show data pertaining to autonomic modulation of pacemaking. In clinical electrophysiology settings isoproterenol, a sympathomimetic drug is used to unmask ectopic sites and we applied that approach to AVRs. In spontaneously beating AVRs, isoproterenol (0.05 µM) potentiated pacemaking and in some AVRs unmasked new ectopic pacemaker sites (Fig. 5A). Multi-electrode mapping in SN and isolated AVR showed isoproterenol induced cycle length shortening to 58.8 ± 1.8% (P < 0.01, n = 3) and 54.3 ± 8.8% (P < 0.001, n = 5) of respective controls (Fig. 5B). In SN isoproterenol acts through β_1_- and β_2_-adrenergic receptors resulting in activation of a G-protein (G_s_) that subsequently activates adenylate cyclase and elevates intracellular levels of cyclic adenosine monophosphate (cAMP)^23^. Direct binding of cAMP to HCN channels results in greater activation of *I*_f_ potentiating pacemaking^24^. cAMP activates protein kinase A, which phosphorylates a number of Ca^2+^- clock proteins amongst others^23^. Action potentials were recorded at the leading AVR pacemaker site and cycle lengths in control and isoproterenol were 551.5 ± 107.3 ms and 304.5 ± 73.3 ms (P < 0.05, n = 4; Fig. 5C), respectively. In quiescent AVRs, application of isoproterenol induced depolarizations that eventually triggered pacemaker action potentials (Fig. 5D). Automaticity in AVR was abolished by the parasympathomimetic, carbachol (0.05 µM; Fig. 5E).

Isoproterenol and carbachol act via adrenergic and muscarinic receptors, respectively. The abundance of mRNA for β_2_-adrenergic receptor (the predominant subtype in SN)^25^ and M_2_-muscarinic receptor in AVR was comparable to SN and significantly higher than ventricular myocardium (Fig. 6A,B). Immunolabelling carried out in tissue sections revealed the pattern of expression of relevant proteins. Using HCN4 as a positive marker for SN and AVR, tissue sections were double labelled with neurofilament-M (NF-M), a marker for sympathetic nerves in the rat heart (Fig. 6C)^26^. AVR sections stained positive for NF-M. Adjacent tissue sections were labelled for β_2_-adrenergic receptor and in the absence of a reliable antibody for the M_2_-receptor, an inward rectifying K^+^ channel, K_ir_3.1 that plays an important role in the parasympathomimetic response (Fig. 6C). AVR myocytes abundantly express β_2_-adrenergic receptors like in SN (Fig. 6D,E). Western blot showed significantly less NF-M in AVR *vs*. SN. Expression levels relative to β-actin was 0.81 ± 0.06 and 0.21 ± 0.05 (P < 0.0001, n = 5, 4), respectively (Fig. 6E). K_ir_3.1 relative abundance was 0.92 ± 0.15 in SN and 0.18 ± 0.05 in AVR (P < 0.01, n = 4).

### Mathematical modelling of AVR ectopy

**Figure 7:**
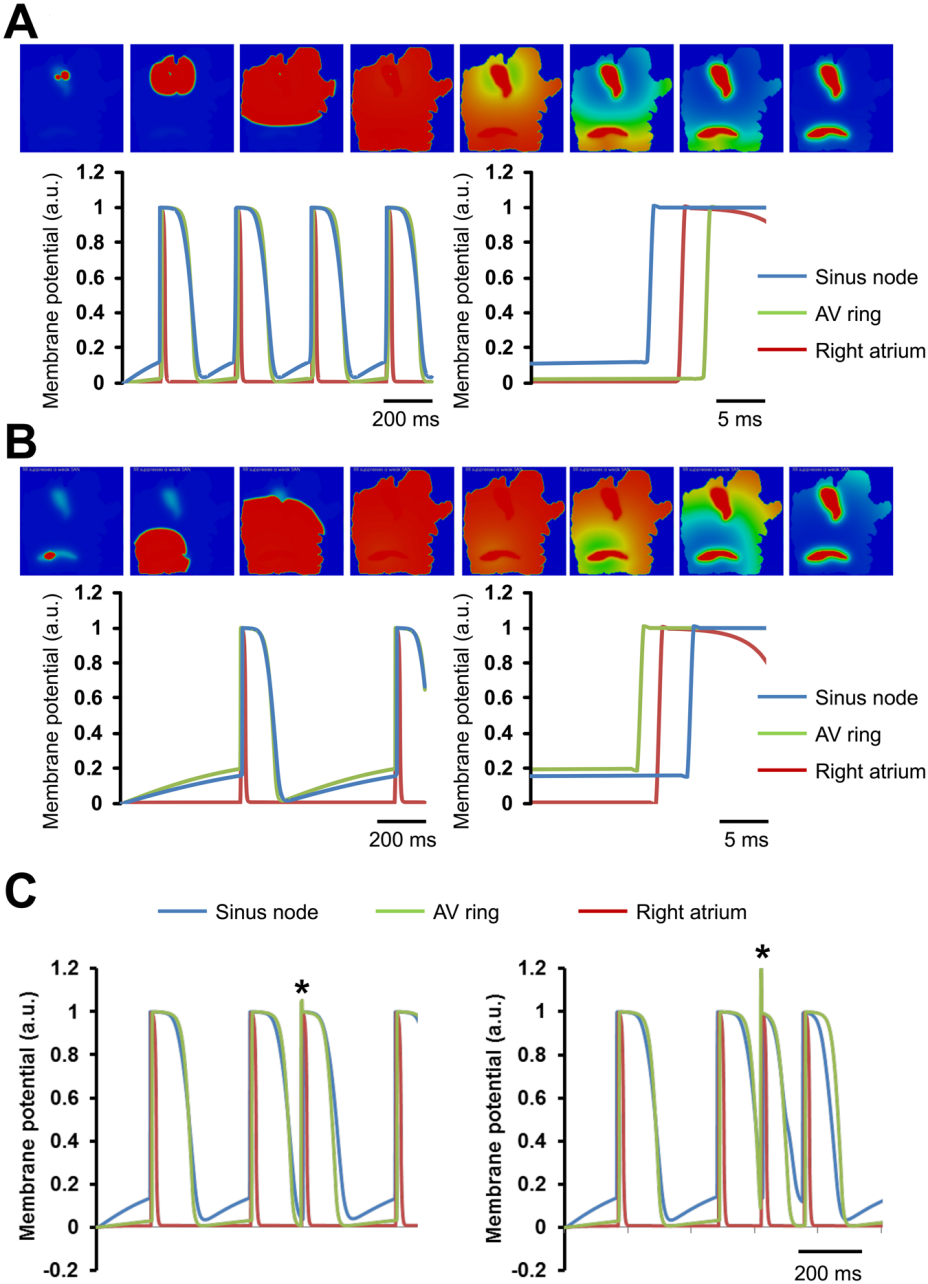
2D computer simulation. (**A**) Normal activation pattern in the right atrium. Top row, sequential electrical propagation maps showing membrane potentials of cells at each location in the 2D sheet. Blue represents resting potential (0 in case of the Fenton Karma model). Red represents an activated action potential. The dominant pacemaker is at the level of the sinus node and overdrives all pacemaker activity in atrioventricular ring (AV ring). Below, single-cell action potential profiles are shown for sinus node (blue trace), right atrium (red trace) and AV ring (green trace). (**B**) In the diseased sinus node, pacemaker activity is elicited in AV ring and propagates through the atrium and supresses sinus node. (**C**) Two examples of premature ectopic beats arising in AV ring (denoted by *) causing suppression of pacemaking in sinus node. Horizontal scale bars represent time in ms.

Simulations were performed to demonstrate how an ectopic beat from AVR may affect SN (Fig. 7) based on the simple Fenton-Karma model. In the free running model, propagating waves commenced at the pacemaking SN (Fig. 7A, top panels). Due to the lower pacing rate assigned to the AVR region, the waves that commenced at SN and propagated into AVR tissue were the primary cause of activation in AVR. The activation of SN was prior to atrial as well as AVR (Fig. 7A, bottom panels). This is illustrated by representative action potentials (Fig. 7A, bottom panels).

Simulation of AVR ectopy is illustrated in Fig. 7B,C. When an ectopic beat was applied at the AVR location at a specific time during the SN cycle length, SN pacemaking was transiently affected. Figure 7B (top panels) show representative frames from the 2D model with wave emerging from AVR location as well as being the cause of SN activation, as opposed to SN activation due to its inherent pacemaking capability. The activation of AVR prior to SN is illustrated by representative action potentials (Fig. 7B, bottom panels). The sequence of tissue activation: AVR followed by atrial followed by SN is presented in Fig. 7B, bottom panels. The ectopy at the AVR promoted extra-systoles as shown in Fig. 7C.

## Discussion

This study has unequivocally demonstrated that AVRs of adult rat hearts can spontaneously generate pacemaker action potentials. We identified multiple commonalities between AVR and SN: action potential phenotype, voltage- and Ca^2+^-clock pacemaker mechanisms, HCN4 expression and autonomic modulation of pacemaking. Computer simulation of action potential propagation in right atrium showed that in the presence of a dominant pacemaker in SN, ectopic pacemaking in AVR is suppressed. Under abnormal circumstances, for e.g. in focal atrial tachycardia, the AVR can become an active ectopic pacemaker.

### Right atrial electrical heterogeneity

Using sharp microelectrode experiments we have confirmed regional variability in right atrial action potential morphology and duration. The only other data regarding regional action potential heterogeneity in atrial myocardium were obtained in rabbit and canine^27–29^. In rat, SN remained the dominant pacemaker and AVRs showed 1:1 action potential conduction consistent with previous reports in rabbit and canine^5,30^. Important aspects of the configuration of conducted AVR action potentials *vs*. those from other atrial regions were: less negative MDP, slow dV/dt_max_, short overshoot and amplitude, consistent with observation in rabbit and canine^5,30,31^. APDs were longer than those in *crista terminalis* and pectinate muscle. MDP is set by the background inward rectifier K^+^ current, *I*_K,1._ Kir2 channels are responsible for *I*_K,1_ and *I*_K,1_ density is highest in ventricular myocytes and low in SN with atrial cells being intermediate, consistent with expression levels of *K*_*ir*_*2* in these tissues^1,32,33^. In rat, *K*_*ir*_*2.1* levels are lower in AVR than in pectinate muscle leading to the less negative MDP in AVR^1^. Action potential upstroke in non-pacemaker atrial cells is fast and brought about by the Na^+^ current, *I*_Na_ and the slower dV/dt_max_ and shorter amplitude in AVRs is likely a result of smaller *I*_Na_. Na_v_1.1 and Na_v_1.5 are the chief Na^+^ channels expressed in rat AV junction^10^. AVRs express lower *Na*_*v*_*1.5* than atrial/pectinate muscle and this is the likely cause for their slower upstroke velocity^1^.

Morphology of the action potential repolarization phase results from the balanced activity of voltage-gated Ca^2+^ (*I*_Ca,L_), transient outward K^+^ current (*I*_to_), and ultra-rapid (*I*_K,ur_), rapid (*I*_K,r_) and slow (*I*_K,s_) delayed rectifier K^+^ currents. In rabbit, APD is much more prolonged in *crista terminalis* than in pectinate muscle, but there is no definite distinctions in *I*_Ca,L_^28^. However, in the canine, APD is long in *crista terminalis* and short in AVR and consistently, *I*_Ca,L_ is greatest in *crista terminalis* and least in AVR^29^. In our investigations of the rat heart, APD measurements in *crista terminalis* are shorter than those of the AVR and AVR myocytes are likely to exhibit smaller *I*_Ca,L_. In rat K_v_1.4, K_v_4.2 and K_v_4.3 channels are known contributors to *I*_to_ and abundance of the corresponding mRNAs in AVR was the same as in atrial muscle^3,34,35^. *K*_*v*_*1.5* is more abundant in AVRs. No significant difference was observed in AVR *vs*. atrial expression for *ERG* and *K*_*v*_*LQT1* responsible for *I*_K,r_ and *I*_K,s_, respectively^3^. One has to bear in mind that although mRNA is a vital determinant of ion channel protein expression, and by extension that of ionic currents, it is not the only determinant and can account for ~40% of the difference^1,36^.

### Ectopic pacemaking in AVR

In right atria, ectopic foci responsible for focal atrial tachycardia do not occur randomly throughout the atria; instead they tend to cluster at characteristic anatomical locations *viz*. along the *crista terminalis*, AVR tissue, and the ostium of the coronary sinus^13,15,37^. In our electrophysiological investigations of right atrium (sans AV node), SN was dominant pacemaker and all other potential pacemakers were discharged by a conducted impulse from SN even before their respective DDs attained threshold. Thus, AVR under normal circumstances is engaged in conducting impulses, but, under an abnormal circumstance i.e. upon detachment of SN, AVR became an actual pacemaker. In the rat right atria, the subsidiary pacemaker in all tissues studied was localised within the AVR. In a goat model of SN disease, ablation of SN unmasked subsidiary pacemakers that were most likely to be localised in the caudal half of the intercaval region (low right atrium)^38,39^. This anatomical location is similar to the “paranodal area” described in the human^40,41^. No evidence of a “paranodal area” with pacemaking capabilities in the rat was observed.

The mean cycle length in AVRs was ~3x larger than SN. 14% of AVR ectopic foci were fast pacemakers with cycle lengths of 190–250 ms, comparable to measurements in SN (170–250 ms). In the remaining 86% of foci, pacemaking was slower than SN. Parameter measurements corresponding to phase 0–4 of action potential provides clues on ionic mechanisms responsible for slower pacemaking in AVR. The phase 4 (i.e. DD) parameters: MDP, TOP and DD amplitude in AVRs were comparable to SN-C. In AVRs it took a long time for the DD to reach TOP from MDP and the DD duration correlated strongly with spontaneous cycle length. Consistently, the DD slope is lowest in AVR. The DD is brought about by a combined effect of voltage- and Ca^2+^-clock pacemaker mechanism^18,20^. The long DD duration in AVR would result from a small *I*_f_ that is likely due to lower HCN4 protein expression^19,42^. Phase 0 of the pacemaker action potential in AVRs characterised by dV/dt_max_, amplitude and peak potential resembled SN-C. Slow dV/dt_max_ in SN-C is brought about by *I*_Ca,L_ and fast *I*_Na_ is absent in SN-C. In SN-P, *I*_Na_ is present and is responsible for the more negative TOP and faster upstroke velocity^43–45^. In AVRs, the less negative TOP (~−40 mV) and low dV/dt_max_ (~5 V/s) can be explained by the absence of *I*_Na_ (the threshold of *I*_Na_ is ~−60 mV); in these cells *I*_Ca,L_ (the threshold of *I*_Ca,L_ is ~−40 mV) is likely responsible for the slow upstroke like in SN-C^43–46^. Action potential repolarization (phase 1–3) in AVR is slower that in SN-C and indicative of smaller outward K^+^ currents (*I*_to_, *I*_K,ur_, *I*_K,r_ and *I*_K,s_) in AVR. The poor expression of K_ir_2 channels in AVR (*vs*. pectinate muscle) and consequently the small *I*_K1_ combined with presence of *I*_f_ can work synergistically and induce automaticity^47^.

### Pacemaker mechanisms

In SN the DD is a result of a synergistic interaction between the voltage-clock and Ca^2+^-clock pacemaker mechanisms. At the beginning of the DD, there is voltage-dependent deactivation of outward K^+^ currents (*I*_K,r_ and *I*_K,s_) and activation of inward currents: *I*_f_, *I*_Ca,T_ and *I*_Ca,L_ amongst others. The Ca^2+^-clock contributes to DD through localised Ca^2+^ release via RYR2. The released Ca^2+^ activates the electrogenic NCX generating an inward current *I*_NaCa_ that imparts a steep, exponential increase to the late phase of DD. SERCA2 refills the sarcoplasmic reticulum with Ca^2+^ and is regulated by phospholamban^18,20,46^. Inhibiting the voltage-clock by blocking *I*_f_ with Cs^+^ and disrupting the Ca^2+^-clock with ryanodine slowed pacemaking in AVR, similar to observations in SN and AVN^18,48^. Expression levels of mRNA transcripts for voltage- and Ca^2+^-clock components in AVR are generally comparable to SN^1,3^. Our investigations revealed no significant difference in expression of Ca^2+^-clock proteins in AVR *vs*. SN; however, HCN4 protein levels were lower and it is likely that *I*_f_ is small, resulting in slower rate of DD and longer cycle lengths in AVR. Our investigations reveal strong voltage-clock involvement in AVR automaticity; however, the degree of cycle length prolongation induced by ryanodine varied considerably and was not always strong. Voltage- and Ca^2+^-clocks interact through some transmembrane Ca^2+^ transporters, such as NCX and voltage-gated Ca^2+^ channels^21,49–52^. Recent evidence suggests a strong cross-talk between *I*_f_ and *I*_NCX_, the functional significance of which may be providing for fail proof pacemaking in the SN^21^. The voltage- and Ca^2+^-clocks mutually entrain to generate pacemaking in the SN and future studies of localised Ca^2+^ release events caused by spontaneous Ca^2+^ cycling processes are needed to provide a convincing case for Ca^2+^-clock involvement in AVR automaticity.

### Autonomic control of ectopic pacemaking

Spatial and temporal patterns of autonomic input, together with the electrophysiological heterogeneity in the atria, provide a substrate for ectopic pacemaking in AVR. The spatial distribution of sympathetic nerves witnessed by NF-M immunolabelling and the cardiomyocyte β_2_-adrenergic receptor expression in AVR is comparable to SN. The rate potentiating effect of isoproterenol, a β_1_- and β_2_-adrenergic receptor agonist, is reminiscent of observations in SN^24,53^, and together these data are indicative of a robust sympathetic innervation in AVRs. Carbachol binds and activates the M_2_-receptor causing activation of *I*_K,Ach_ and bringing about a more negative MDP, thus slowing pacemaking^53^. K_ir_3.1 is partly responsible for *I*_K,Ach_ and AVRs express low levels of this important protein. A prolonged APD (with reduced *I*_to_) and reduced *I*_K,Ach_ is likely to result in increased ectopic activity as evidenced elsewhere in the posterior left atrial myocardium^54^.

### Computer simulations

It is well known that ectopy in the heart may affect SN pacemaking^55^. In our model, a single ectopic beat from the AVR affected SN pacemaking transiently. Our findings are in line with those of Ai *et al*. who observed similar transient overdrive suppression behaviour in a nodal pacemaker cell^56^. Varying of SN-atrial tissue junction strengths or a change of ectopic beat pacemaking is known to affect this transient suppression of pacemaking^57^. In contrast to our experimental results, the full ranges of SN and AVR pacemaking have not been included in our computer model that is based on the simple Fenton-Karma model. Furthermore, ionic mechanisms that may affect pacemaking and atrial tissue electrophysiological properties have been excluded in the modelling for simplicity. We appreciate that use of ionically detailed electrophysiology will also permit the inclusion of further accurate tissue conduction properties. Notwithstanding the limitations, our current approach demonstrated that AVR ectopy can deleteriously affect physiological pacemaking in the SN.

## Methods

All animal care and usage was according to standards and practices approved by the University of Manchester Animal Welfare and Ethical Review body and in accordance with the Animals (Scientific procedures) Act, 1986. Wistar rats (male, 275–375 g) were killed by percussive blow to head followed by cervical dislocation. Animal experiments conformed to guidelines from Directive 2010/63/EU of European Parliament on protection of animals used for scientific purposes. Data are presented as mean ± s.e.m. Unpaired t-test or one-way ANOVA followed by Tukey’s multiple comparisons post-test was conducted and differences were considered significant at P < 0.05.

### Tissue preparation for electrophysiology

Hearts were cleared of blood by retrograde aortic perfusion (~10 min) in Langendorff setup with oxygenated (95% O_2_-5% CO_2_) Tyrode’s solution (in mM: NaCl 120.3, KCl 4.0, CaCl_2_ 1.2, MgSO_4_ 1.3, NaH_2_PO_4_ 1.2, NaHCO_3_ 25.2, glucose 11, pH 7.4, 37 °C). A longitudinal incision was made along the atrial septum and the right atrium, intact with the basal segment of right ventricle, was dissected and pinned onto silicone rubber strips in a thermostatically controlled 5 ml tissue chamber perfused (20 ml/l) with pre-warmed Tyrode’s bubbled with 95% O_2_-5% CO_2_. Bath solution temperature was controlled at 37 ± 0.2 °C, and after ~20 min equilibration, electrophysiological investigations were performed on the endocardial surface. To study isolated AVR, SN was excised by cutting along the length of the *crista terminalis*. The location of the leading pacemaker site was quickly located by multi-electrode array extracellular electrical mapping, arrays were removed and intracellular action potentials recorded by sharp microelectrode technique.

### Extracellular electrical mapping

For extracellular mapping of electrical impulse propagation in right atrial preparations, a rectangular (4.5 × 3.7 mm) 8 × 8 electrode array (0.2 mm electrode diameter, 0.6 mm inter-electrode distance) was used. For studies on isolated AVRs, a smaller (4.5 × 1.5 mm) 6 × 10 electrode array (0.25 mm electrode diameter, 0.2 mm inter-electrode distance) was used. Electrode arrays were handcrafted in our laboratory. Signals acquired at 1.5 kHz were amplified (100×) and digitized with PXI-6031E cards (National Instruments, U.S.A.). Signals were not filtered. Data were acquired and analysed using custom written software. The moment of maximal negative rate of change of potential in the extracellular electrogram was automatically detected, saved as time of local activation, and used to draw activation maps in MATLAB (MathWorks, U.S.A.).

### Intracellular action potential recordings

Intracellular action potentials were recorded in tissue preparations using sharp microelectrodes. Microelectrodes drawn from borosilicate glass capillaries (1.2 mm outer and 0.6 mm internal diameter; Hilgenberg, Germany) in Narishige PE-2 puller (Narishige, Japan) were back-filled with 3 M KCl and inserted into an Ag-AgCl pellet holder (Model E45P-M15N, Harvard Apparatus, U.K.) prefilled with 3 M KCl. Typical microelectrodes had electrical resistance of 20–40 MΩ. An Ag-AgCl disk electrode (Model E242, Harvard Apparatus, U.K.) served as bath ground return.

**Figure 8:**
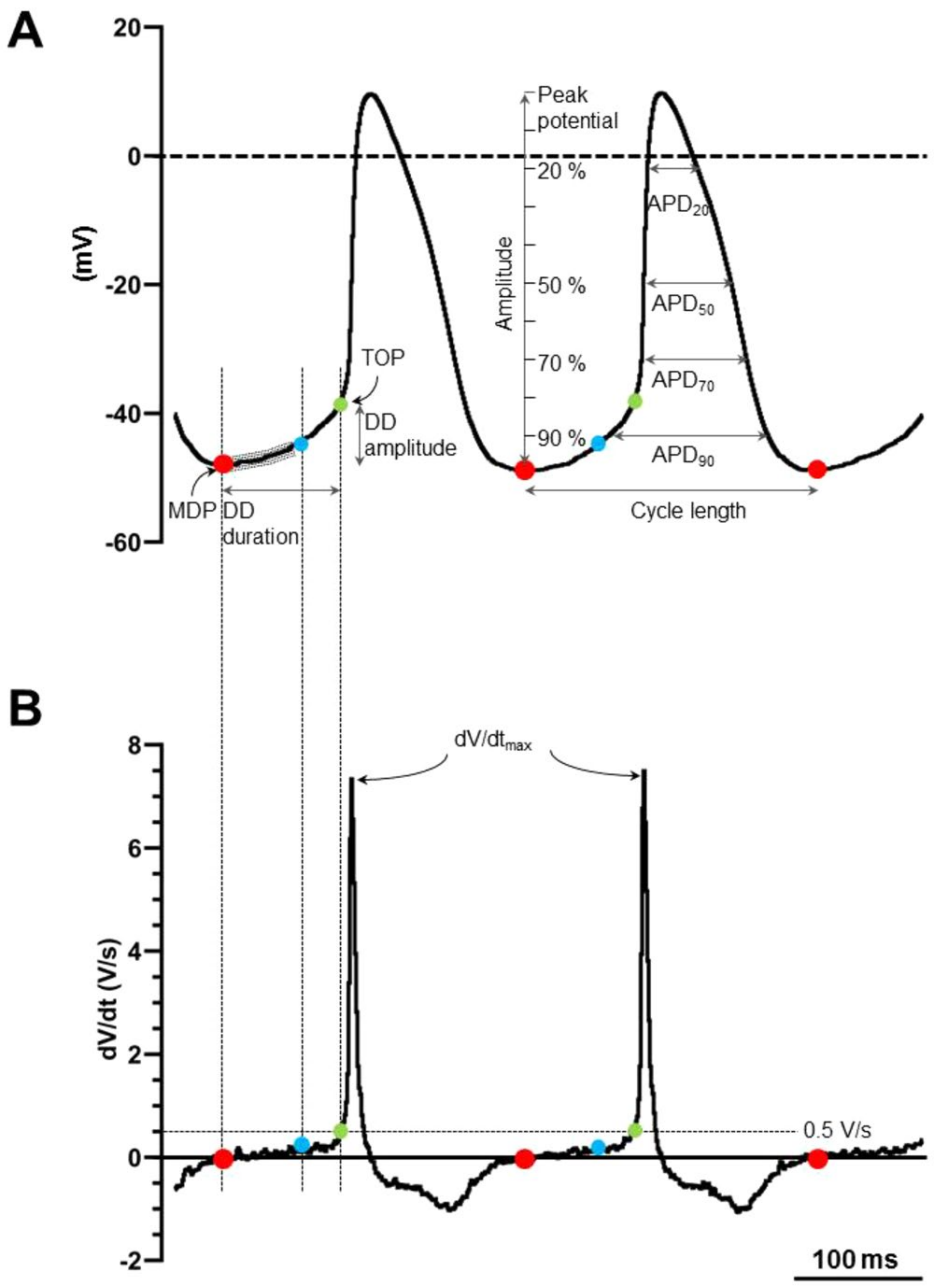
Action potential parameters and measurements. Representative action potential record from the leading pacemaker site in a rat sinus node preparation (mV scale in **A**) and corresponding first time derivative dV/dt (mV/s scale in B) are shown. The maximum diastolic potential (MDP, filled red circle), take-off potential (TOP, filled green circle), diastolic depolarization (DD) duration (time interval between MDP and TOP) and DD amplitude (difference between TOP and MDP) are indicated (**A**). The DD slope for each action potential record was calculated as the mean of dV/dt values (**B**, mV/s) in the early two-thirds fraction (shaded interval between red and blue circles in panel A) of the DD duration. Peak potential was noted as the maximum potential reached (**A**, mV), amplitude calculated as difference between MDP and peak potential and maximum upstroke velocity was the maximum dV/dt value reached (**B**, mV/s). The time dependent variables of the action potential *viz*. cycle length and APD at 20%, 50%, 70% and 90% repolarization was measured as indicated.

In right atrial preparations, intracellular action potentials were recorded from SN (centre and periphery), intercaval region, area immediately surrounding the leading pacemaker site, *crista terminalis*, pectinate muscle and AVR. Voltage measurements made relative to bath potential at 0.1 ms intervals (10 kHz) were passed through 10-kHz low-pass Bessel filter, amplified 10× (GeneClamp 500 amplifier, Molecular Devices, U.S.A.), digitized (Digidata 1440 A, Molecular Devices, U.S.A.) and stored on computer for later analysis. Action potentials were recorded as continuous traces using WinEDR V3.3.6 software (Dr J. Dempster, University of Strathclyde, Glasgow, UK). Series of five consecutive action potentials per impalement were exported in the Axon binary file format and later imported into LabChart V8 (ADInstruments, Australia) for parameter measurements. Corresponding parameter measurements from the five consecutive action potentials were averaged per individual impalement. For pacemaker action potentials, diastolic depolarization parameters were also measured. For this, raw action potential records were digitally smoothed in LabChart using the 10-point adjacent averaging (decimation) smoothing function and the first order time derivative (slope, dV/dt) was calculated using the derivative function (window width 3 points). The following action potential parameters were measured (also see Fig. 8):

i). Cycle length (ms), time interval between consecutive action potential peaks.
ii). Maximum diastolic potential (MDP, mV), most negative membrane potential reached between consecutive action potential peaks.
iii). Take-off potential (TOP, mV), voltage measured when dV/dt reached an empirically determined threshold of 0.5 V/s. At this threshold, dV/dt changed abruptly during the transition from phase 4 to phase 0 of the SN and AVR pacemaker action potential, irrespective of the spontaneous beating rate (Fig. 8).
iv). Diastolic depolarization (DD), phase of the action potential in the interval between MDP and TOP. DD duration (ms) was calculated as time interval between MDP and TOP and DD amplitude (mV) was the difference between TOP and MDP. Slope of DD (DD slope, mV/s) was calculated as mean value of dV/dt data points during the first two-thirds of the DD. During this period the slope was roughly constant and could be represented by a liner function.
v). Peak potential (mV), maximum membrane potential at the action potential peak.
vi). Maximum upstroke velocity (dV/dt_max_, V/s), maximum value for the first derivative of transmembrane voltage between MDP and peak potential.
vii). Amplitude (mV), action potential height measured at the peak relative to MDP
viii). Action potential duration (APD_R_, ms), where R is one of 10, 20, 50, 70 or 90, was calculated as the shortest time between crossings of MDP + (100 − R)/100*Amplitude on either side of the peak (see Fig. 8).

Action potentials were classified into categories based on anatomical localization of the microelectrode impalement. SN and AVR pacemaker action potentials were identified by their prominent phase 4 DD. Using a multi-electrode array electrical mapping system it was possible to localise the leading pacemaker site to a small zone (<0.4 mm^2^) in SN and in isolated AVRs. In SN, pacemaker action potentials with dV/dt_max_ values <10 V/s were classified as central or leading (SN-C) and those with >10 V/s were classified peripheral or follower action potentials (SN-P).

### Quantitative PCR

Freshly dissected right atrial preparations from 12 hearts were covered in OCT compound (VWR International, U.S.A.) and flash frozen with liquid nitrogen cooled 2-methylbutane (Sigma-Aldrich, U.S.A.). Abundance of mRNA transcripts was measured by quantitative PCR (qPCR) as described previously^3^. Briefly, frozen tissues sectioned at 50 µm thickness in −20 °C CM3050s cryostat (Leica, Germany) were transferred onto ultraviolet light treated PEN‐membrane slides (Leica Microsystems, Germany) and stored at −80 °C. SN, right atrial and right AVR samples were collected by laser microdissection (Leica LMD6000 system, Leica Microsystems, Germany), RNA extracted with RNAqueous®-Micro kit (Applied Biosystems, U.S.A.) and treated with DNase 1. Total RNA concentration was measured using Nanodrop ND1000 spectrophotometer (Thermo Fisher Scientific, U.S.A.) and RNA integrity analysed in Agilent Bioanalyzer (Agilent Technologies, U.S.A.). Total RNA was reverse transcribed to produce cDNA using high capacity RNA-to-DNA Master Mix (Applied Biosystems, U.S.A.). Abundance of transcripts was measured using Taqman low density array microfluidic cards (Applied Biosystems, U.S.A.) for qPCR. Data was analysed using RQ manager (Applied Biosystems, U.S.A.) and expression levels calculated using the ΔCt method with 18 S as housekeeper/reference transcript. Outliers relating to biological replicates were removed using StatMiner (Integromics, Spain) via the median of absolute deviation analysis, with samples with >3 flags being removed. Barcharts show 2^−ΔCt^ mean ± s.e.m. Using StatMinerTM a moderated t-test (limma parametric) was performed on ΔΔCt values and as a correction for multiple comparisons a Benjamini–Hochberg adjustment for false discovery rate (FDR) was calculated. FDR <0.2 was considered significant.

### Immunohistochemistry and western blot

Immunolabelling and Western blot was performed as described by us previously^18,58^. For immunohistochemistry, four hearts dissected in oxygenated Tyrode’s were flash frozen in liquid nitrogen. For Western blot, five SN and AVR biopsies were frozen in liquid nitrogen. Samples were stored at 80 °C. Details of primary and secondary antibodies are available in data Supplement Table 1. Immunolabelled tissue sections were imaged on laser scanning confocal microscope (LSM5, Carl Zeiss, Germany) using Pascal software (Carl Zeiss, Germany). Western blot membranes were imaged in ChemiDoc MP imaging system (Bio-rad Laboratories, U.S.A.) and chemiluminescent signal intensities were normalised to the corresponding β-actin intensity.

### Computer simulations

Conditions favouring ectopic foci in AVR were assessed by computer simulations. A 2D sheet model consisting of SN, atrial, and AVR tissue was constructed and the purpose was to demonstrate that AV ectopy affects SN pacemaking.

Cell model: The established Fenton-Karma cell model was modified to simulate pacemaker action potentials with cycle lengths of ~200 ms^59,60^. AVR cell model was given the same pacemaking properties as SN cell model. Atrial cell model was constructed to permit simulating a short action potential.

Spatial model consisting of three tissue types: A spatial model consisting of approximately 150 (x) x 150 (y) cells was constructed. Inter-cellular distance was taken to be 0.1 mm. Based on expert opinion, a region of the model was assigned SN properties and another region was given AVR properties. Electrical waves commenced either at SN, AVR, or both SN and AVR sites depending on initial conditions. The rest of the electrically active tissue was assumed to be atrial tissue. Numerical solutions were obtained using our parallel mono-domain equations solver for electrical wave propagation in the heart^60^. Tissue diffusion was set to reproduce atrial wave speeds of 0.6 m/s. Simulations were performed for 10 s of electrical activity, and the final 2 s was recorded. Simulations consisted of either free running model where SN paced surrounding tissue, or when erratic wave initiating stimuli were applied at the AVR site.

Numerical methods: A recently developed implicit finite difference solver^60^ was used to obtain accurate numerical solutions efficiently.

## Supplementary information

Logantha et al Sup Info
