# Supplementary material for: Sinus node-like pacemaker mechanisms regulate ectopic pacemaker activity in the adult rat atrioventricular ring
Source: Sci Rep. 2019 Aug 13;9:11781. doi: 10.1038/s41598-019-48276-0 (PMC6692414; doi:10.1038/s41598-019-48276-0)

Supplementary Information

Sinus node-like pacemaker mechanisms regulate ectopic pacemaker activity in the adult rat atrioventricular ring

Logantha: Ectopic pacemaking in the atrioventricular rings

Sunil Jit R.J. Logantha, PhD[1*]; Sanjay R. Kharche, PhD[1, 2]; Yu Zhang, MSc[1]; Andrew J. Atkinson, MPhil[1]; Guoliang Hao, PhD[1]; Mark R. Boyett[1], PhD; Halina Dobrzynski, PhD[1*]

[1]Division of Cardiovascular Sciences, Faculty of Biology, Medicine and Health, University of Manchester, Manchester, United Kingdom.

[2]Lawson's Health Research Institute, Department of Medical Biophysics, University of Western Ontario, London ON, Canada.

[*]Corresponding authors

Correspondence address: Dr. Sunil Jit Logantha and Dr. Halina Dobrzynski; Division of Cardiovascular Sciences, University of Manchester, 3rd Floor Core Technology Facility Building, 46 Grafton Street, Manchester M13 9NT, United Kingdom.

Email: sunil.logantha@manchester.ac.uk and halina.dobrzynski@manchester.ac.uk

Phone: +44-161-2751207 and +44-161-2751182

Fax: (+)44-161-2651183

Supplement Table 1. Action potential heterogeneity in right atrium.

Action potentials were recorded in spontaneously beating right atrial preparations with intact sinus node and right atrioventricular (AV) ring. Parameters measured in the intercaval region (between sinus node and atrial septum), surrounding the sinus node (surrounding the leading pacemaker site), *Crista terminalis*, pectinate muscle and right AV ring are shown in columns a-e, respectively. Data are presented as mean ± SEM and 'n' refers to number of cells. Significant differences (P<0.05) *vs*. corresponding values in other atrial regions (i.e. columns) is shown in brackets.

	Column a<br>Intercaval<br>Region	Column b<br>Surrounding<br>sinus node	Column c<br>*Crista*<br>*Terminalis*	Column d<br>Pectinate<br>Muscle	Column e<br>Right<br>AV ring
Parameters	n = 21 cells<br>10 hearts	n = 63 cells<br>16 hearts	n = 29 cells<br>11 hearts	n = 37 cells<br>18 hearts	n = 50 cells<br>16 hearts
Cycle length<br>(ms)	196.7 ± 3.5	196.5 ± 2.8	208.5 ± 4.1	204.1 ± 3.7	202.3 ± 2.9
MDP<br>(mV)	-81.2 ± 1.3<br>(e)	-79.7 ± 0.7<br>(e)	-78.9 ± 0.9<br>(e)	-77.4 ± 0.8<br>(e)	-73.4 ± 1<br>(a, b, c, d)
dV/dt_{max}<br>(V/s)	166.1 ± 8.1<br>(e)	165.2 ± 4.5<br>(e)	171.7 ± 8.1<br>(e)	146.4 ± 6.4<br>(e)	107.9 ± 6.9<br>(a, b, c, d)
Peak potential<br>(mV)	14.5 ± 1.6<br>(e)	16.1 ± 0.9<br>(e)	18.8 ± 1.4<br>(d, e)	13.3 ± 1.2<br>(c, e)	7.6 ± 1.2<br>(a, b, c, d)
Amplitude<br>(mV)	96.2 ± 2.1<br>(e)	96.7 ± 1.1<br>(d, e)	97.7 ± 1.4<br>(e)	91 ± 1.3<br>(b, e)	81.4 ± 1.7<br>(a, b, c, d)
APD_{10}<br>(ms)	2.6 ± 0.3<br>(e)	2.4 ± 0.3<br>(e)	1.8 ± 0.2<br>(e)	1.8 ± 0.1<br>(e)	5 ± 0.6<br>(a, b, c, d)
APD_{20}<br>(ms)	5.9 ± 0.6	5.1 ± 0.5<br>(e)	4.1 ± 0.4<br>(e)	3.6 ± 0.2<br>(e)	8.9 ± 0.9<br>(b, c, d)
APD_{30}<br>(ms)	10.1 ± 0.9	8.6 ± 0.6<br>(e)	7.1 ± 0.7<br>(e)	5.9 ± 0.4<br>(e)	13.4 ± 1.2<br>(b, c, d)
APD_{50}<br>(ms)	22.3 ± 1.5<br>(c, d)	18.9 ± 1<br>(d, e)	15 ± 1.3<br>(a, e)	11.5 ± 0.8<br>(a, b, e)	24.2 ± 1.5<br>(b, c, d)
APD_{70}<br>(ms)	42.9 ± 2.1<br>(c, d)	36.8 ± 1.6<br>(c, d)	27.6 ± 2.2<br>(a, b, e)	20.5 ± 1.3<br>(a, b, e)	40.1 ± 1.8<br>(c, d)
APD_{80}<br>(ms)	56 ± 2.4<br>(c, d)	48 ± 1.9<br>(c, d)	36.1 ± 2.8<br>(a, b, e)	27.1 ± 1.5<br>(a, b, e)	50.9 ± 2<br>(c, d)
APD_{90}<br>(ms)	74.8 ± 2.7<br>(c, d)	64.8 ± 2.1<br>(c, d)	50 ± 3.6<br>(a, b, e)	39.1 ± 1.8<br>(a, b, e)	68.4 ± 2.4<br>(c, d)

Supplement Table 2. List of primary and secondary antibodies.

	Protein	Host	Type	Dilution	Cat. No. and supplier
**Primary antibodies**					
Immunohistochemistry and Western blot	HCN4	Rabbit	Polyclonal IgG	1:50	APC-052, Alomone labs
	RyR2	Mouse	Monoclonal IgG	1:100	MA3-916, Thermo Fisher Scientific
	SERCA2	Rabbit	Polyclonal IgG	1:100	ACP-012, Alomone labs
	β-actin	Mouse	Monoclonal IgG	1:200	A5441, Sigma-Aldrich
	NF-M	Mouse	Monoclonal IgG	1:100	G9670, Sigma-Aldrich
	β_2 adrenergic receptor	Mouse	Monoclonal IgG	1:50	ab182136, abcam
	$K_{ir}3.1$	Rabbit	Polyclonal IgG	1:50	APC-005, Alomone labs
**Secondary antibodies**					
Immunohistochemistry	Cy3 conjugate	Goat	anti-rabbit IgG	1:400	AP132C, Millipore
	FITC conjugate	Goat	anti-mouse IgG	1:100	AP124F, Millipore
	FITC conjugate	Goat	anti-rabbit IgG	1:100	AP132F, Millipore
Western blot	Horseradish Peroxidase conjugate	Goat	anti-rabbit IgG	1:2000	111-035-144, Jackson Immuno Research labs
	Horseradish Peroxidase conjugate	Goat	anti-mouse IgG	1:1000	62-6520, Thermo Fisher scientific

Supplement Fig.1: Correlation between pacemaker action potential parameter measurements.

A-D, correlation plots are shown for sinus node (left plots) and AV ring (AVR; right plots). Measurements are form 21 sinus node-centre (SN-C; black squares), 19 sinus node-periphery (SN-P, grey squares) and 50 AV ring (AVR; white squares) myocytes. Best-fit trend lines were fitted by linear regression and the results of linear regression are shown as an inset.

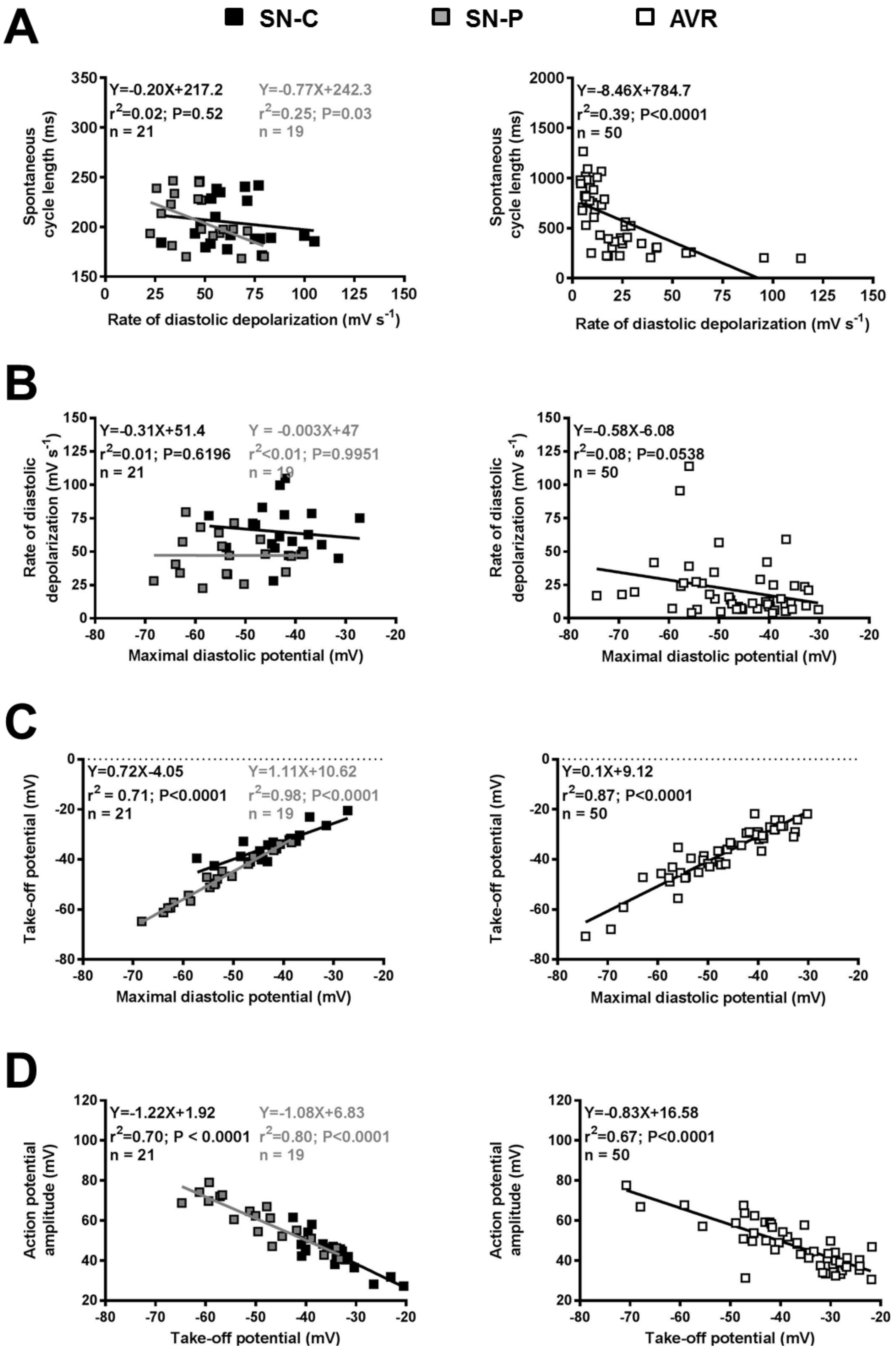
A
SN-C
SN-P
AVR
Y=-0.20X+217.2
r2=0.02; P=0.52
n = 21
Y=-0.77X+242.3
r2=0.25; P=0.03
n = 19
Spontaneous cycle length (ms)
Rate of diastolic depolarization (mV s-1)
Y=-8.46X+784.7
r2=0.39; P<0.0001
n = 50
B
Y=-0.31X+51.4
r2=0.01; P=0.6196
n = 21
Y = -0.003X+47
r2<0.01; P=0.9951
n = 19
Rate of diastolic depolarization (mV s-1)
Maximal diastolic potential (mV)
Y=-0.58X-6.08
r2=0.08; P=0.0538
n = 50
C
Y=0.72X-4.05
r2 = 0.71; P<0.0001
n = 21
Y=1.11X+10.62
r2=0.98; P<0.0001
n = 19
Take-off potential (mV)
Maximal diastolic potential (mV)
Y=0.1X+9.12
r2=0.87; P<0.0001
n = 50
D
Y=-1.22X+1.92
r2=0.70; P < 0.0001
n = 21
Y=-1.08X+6.83
r2=0.80; P<0.0001
n = 19
Action potential amplitude (mV)
Take-off potential (mV)
Y=-0.83X+16.58
r2=0.67; P<0.0001
n = 50

Supplement Fig.2: Unprocessed original scans of Western blot gel for β-actin.

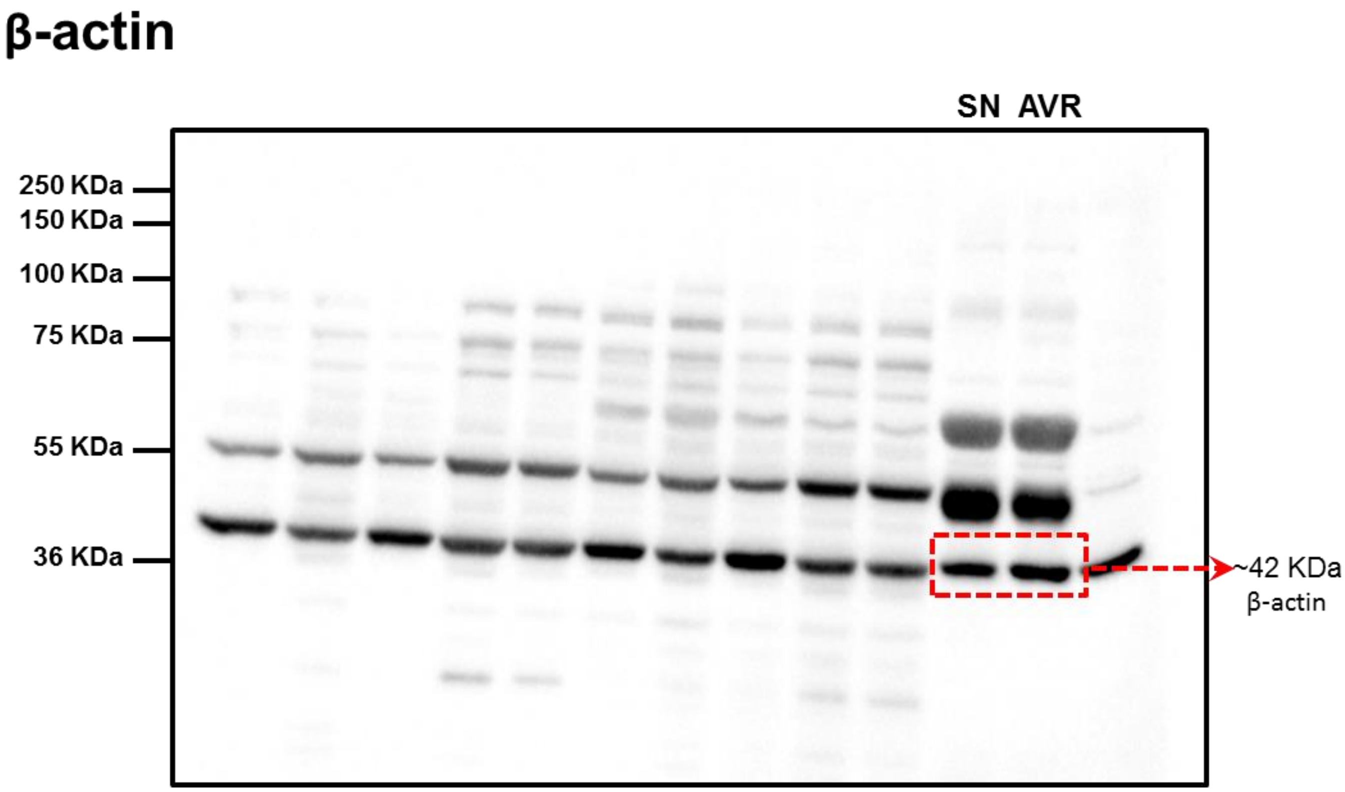

Supplement Fig.3: Unprocessed original scans of Western blot gel for HCN4.

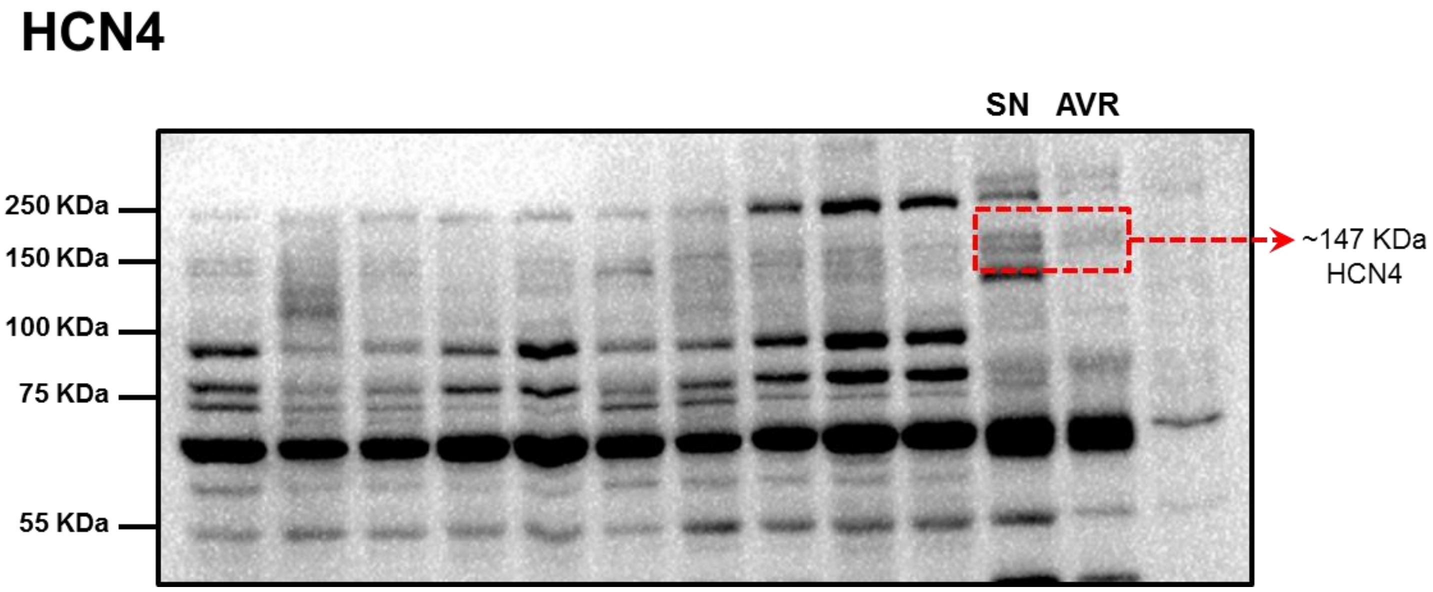

Supplement Fig.4: Unprocessed original scans of Western blot gel for SERCA2a.

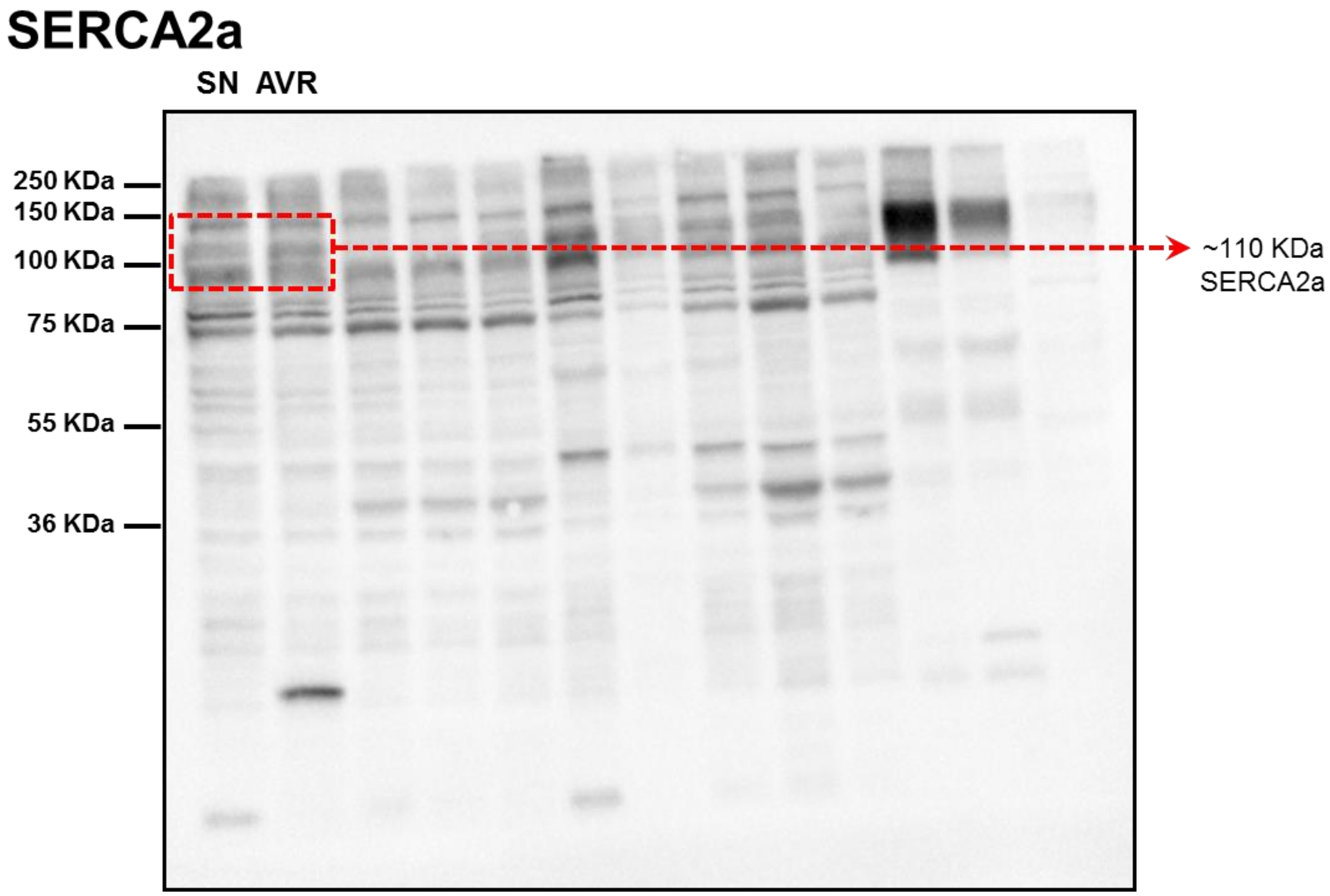

Supplement Fig.5: Unprocessed original scans of Western blot gel for β_2-ADR.

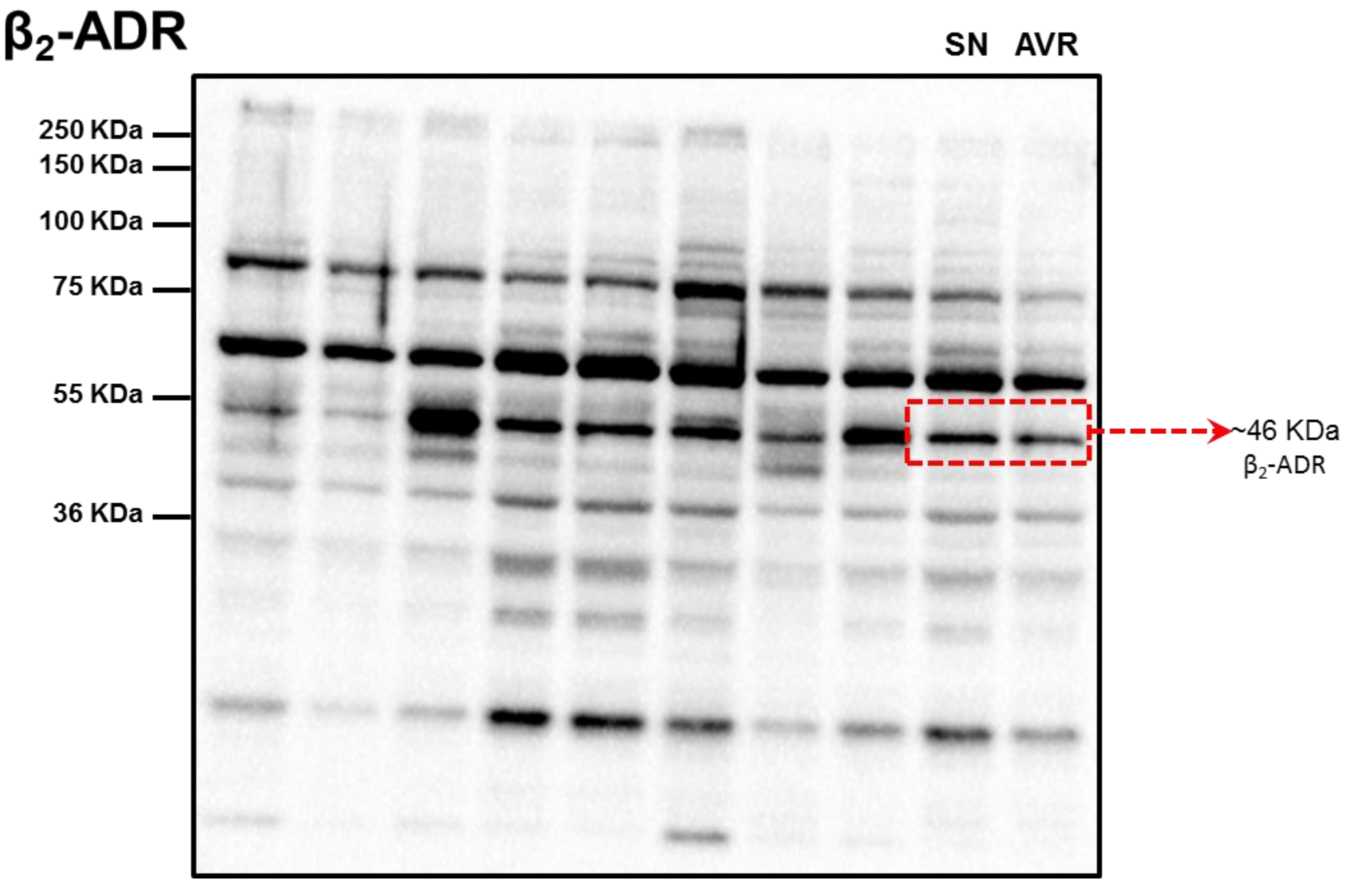

Supplement Fig.6: Unprocessed original scans of Western blot gel for NF-M.

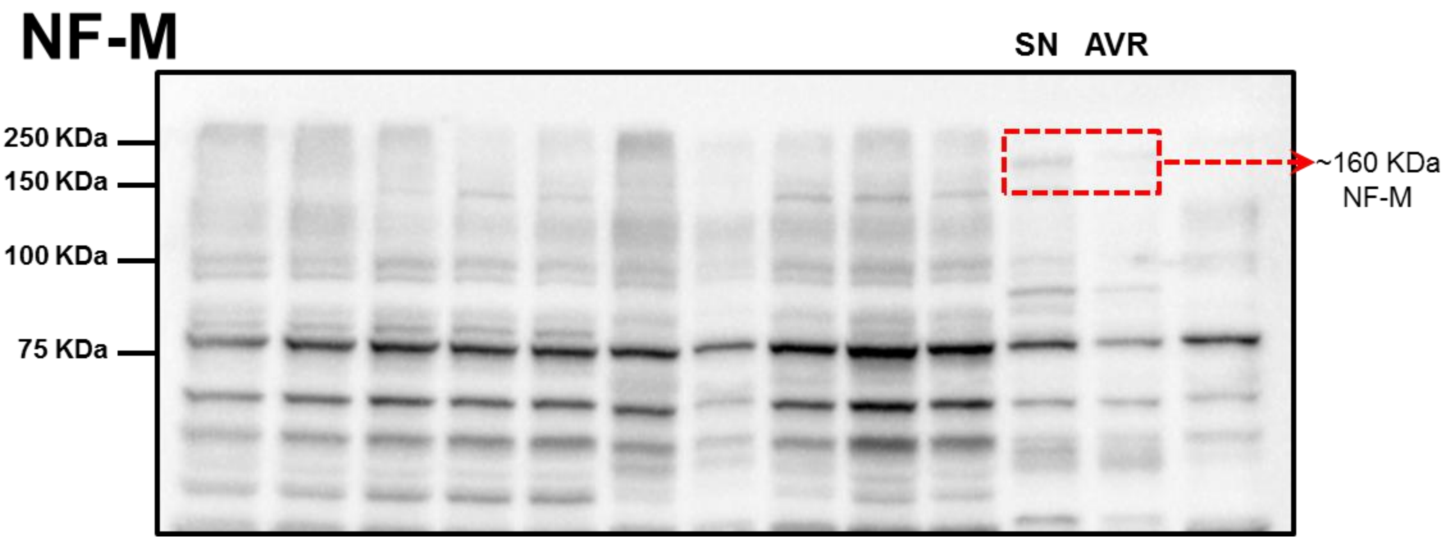

Supplement Fig.7: Unprocessed original scans of Western blot gel for $K_{ir}3.1$.

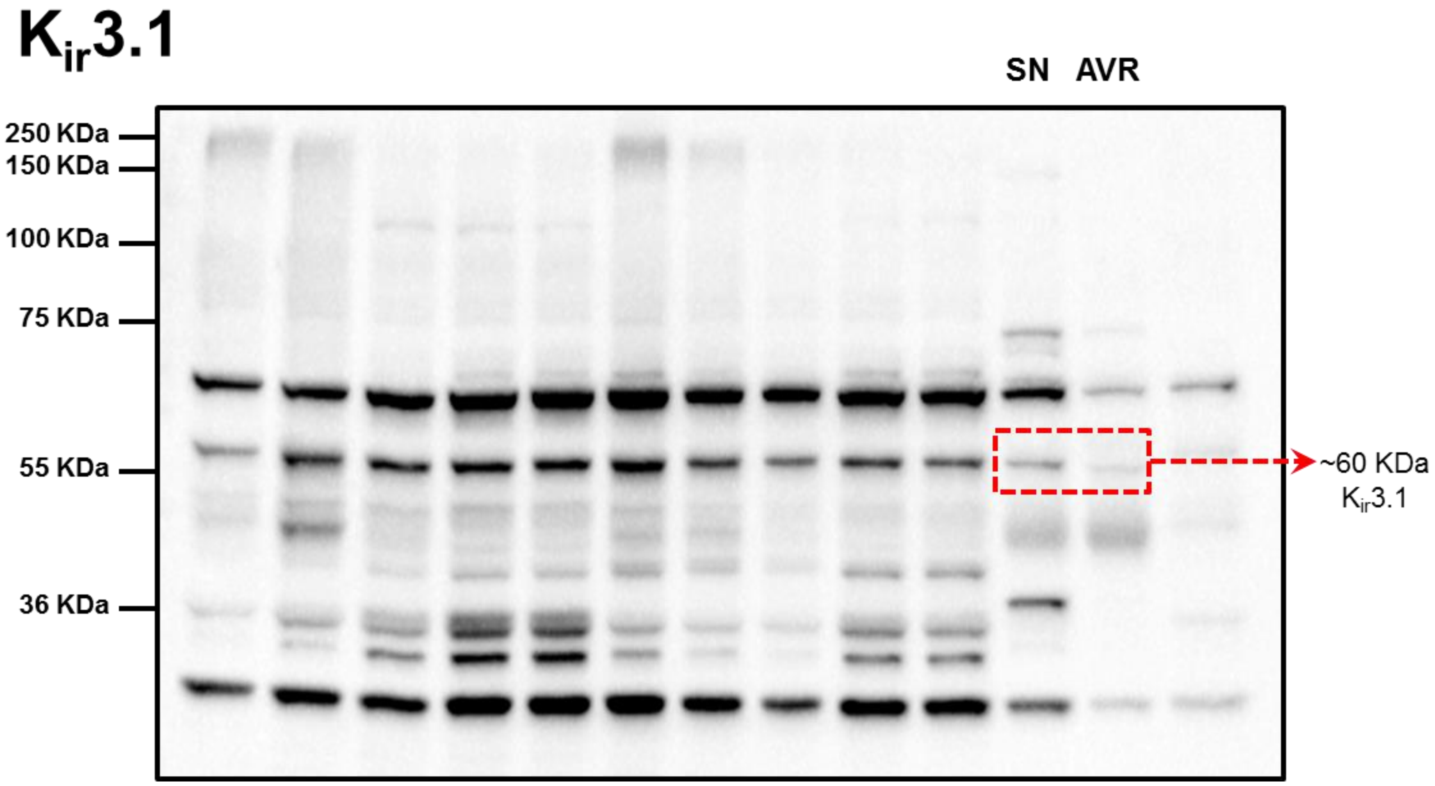

Supplement: Supplementary file 1 — Logantha et al Sup Info [file 41598_2019_48276_MOESM1_ESM.pdf]
